# Comparative anatomy and metabolic profiles of brown and white adipose tissue in humans

**DOI:** 10.3389/fendo.2026.1821542

**Published:** 2026-04-28

**Authors:** Jesus Villarreal, Ancha Baranova

**Affiliations:** 1 Department of Health and Behavioral Sciences, Texas A&M University–San Antonio, San Antonio, TX, United States; 2 School of Systems Biology, George Mason University, Fairfax, VA, United States; 3 Research Centre for Medical Genetics, Moscow, Russia

**Keywords:** adipose imaging, adipose tissue plasticity, beige adipocytes, brown adipose tissue, metabolic regulation, obesity, PET-CT, thermogenesis

## Abstract

Adipose tissue is a dynamic metabolic organ that plays a central role in energy homeostasis, endocrine signaling, and thermoregulation (Forner et al., 2009). Traditionally, adipose tissue has been classified into two major types: white adipose tissue (WAT), which primarily stores energy as triglycerides, and brown adipose tissue (BAT), which dissipates energy as heat through non-shivering thermogenesis. While WAT is widely distributed throughout the human body, BAT is more abundant in small mammals and infants but remains present and metabolically active in specific regions of adult humans. Recent molecular biology discoveries and imaging methods have reshaped the physiological status of BAT and recognized the presence of beige or brite adipocytes, which develop in WAT depots and display thermogenic potential. This led to renewed attention on adipose tissue plasticity and how it is relevant for metabolic health, obesity, and associated metabolic disorders. This narrative review compares the anatomical distribution, cellular morphology, developmental origins, and physiological functions of WAT and BAT in humans. It also summarizes imaging modalities used to identify metabolically active adipose tissue and discusses emerging concepts such as adipose browning, endocrine signaling, and therapeutic activation of thermogenic fat.

## Introduction

Adipose tissue is an integral component of the human body and performs a wide range of physiological functions (1). Beyond serving as a reservoir for energy storage, adipose tissue contributes to mechanical protection of internal organs, thermal insulation, and endocrine regulation (2). Adipose depots give rise to hormones, cytokines, and signaling molecules that affect appetite, insulin sensitivity, inflammation, and metabolic homeostasis.

Typically, adipose tissue has been classified into two main types: white adipose tissue (WAT) and brown adipose tissue (BAT). Both WAT and BAT are involved in balancing energy; however, they differ when it comes to structure, metabolism, location, and physiology based on the composition and function of their components. WAT primarily stores excess energy in the form of triglycerides, whereas BAT dissipates stored energy as heat through non-shivering thermogenesis (3).

BAT is especially abundant in small mammals and in newborn human infants, where it plays a vital role in maintaining body temperature (4). Since infants have a relatively large surface-area-to-volume ratio, they are more susceptible to heat loss. Due to insufficient musculature for effective shivering, they depend heavily on BAT-mediated thermogenesis to live in cooler conditions. BAT was once thought, for many decades, to be missing or metabolically insignificant in human adults.

Nevertheless, recent developments in imaging methods (e.g., positron emission tomography combined with computed tomography, PET-CT) have revealed metabolically active BAT depots in adult subjects. These findings have revolutionized the concept of adipose tissue biology and rekindled interest in the contribution of thermogenic fat to human metabolism (5).

In addition to classic BAT, a third adipocyte type called beige or brite adipocytes has been reported within WAT. They can also be induced under specific physiological conditions (e.g., cold exposure, adrenergic stimulation), which results in thermogenic and classical brown adipocyte phenotypes. This phenomenon, known as adipose tissue “browning, ” illustrates the remarkable plasticity of adipose tissue.

Understanding the structural, developmental, and physiological differences among WAT, BAT, and beige adipocytes is essential for advancing knowledge of metabolic regulation. These insights may ultimately contribute to therapeutic strategies targeting obesity, metabolic syndrome, and related disorders.

Since the discovery of metabolically active BAT in adult humans, research has increasingly focused on understanding how BAT can be activated or expanded to improve metabolic health. Early landmark studies revealed that cold exposure activates BAT in adults (6, 7), and subsequent research has further clarified its metabolic significance.

## Literature review search strategy

Literature was reviewed using PubMed, Google Scholar, and Scopus databases that are relevant for comparative anatomy of adipose tissue, metabolic regulation, and thermogenesis in adult humans. The search included keywords such as “brown adipose tissue”, “white adipose tissue”, “beige adipocytes”, “thermogenesis”, “adipose tissue plasticity”, and “metabolic regulation.” The search primarily consisted of studies that were published between the years 2000 to 2024. Furthermore, we focused on recent advances and discoveries within the last five years. Additional references were identified through citation tracking of relevant articles.

## Anatomy and distribution of adipose tissue

### White adipose tissue

**Figure 1:**
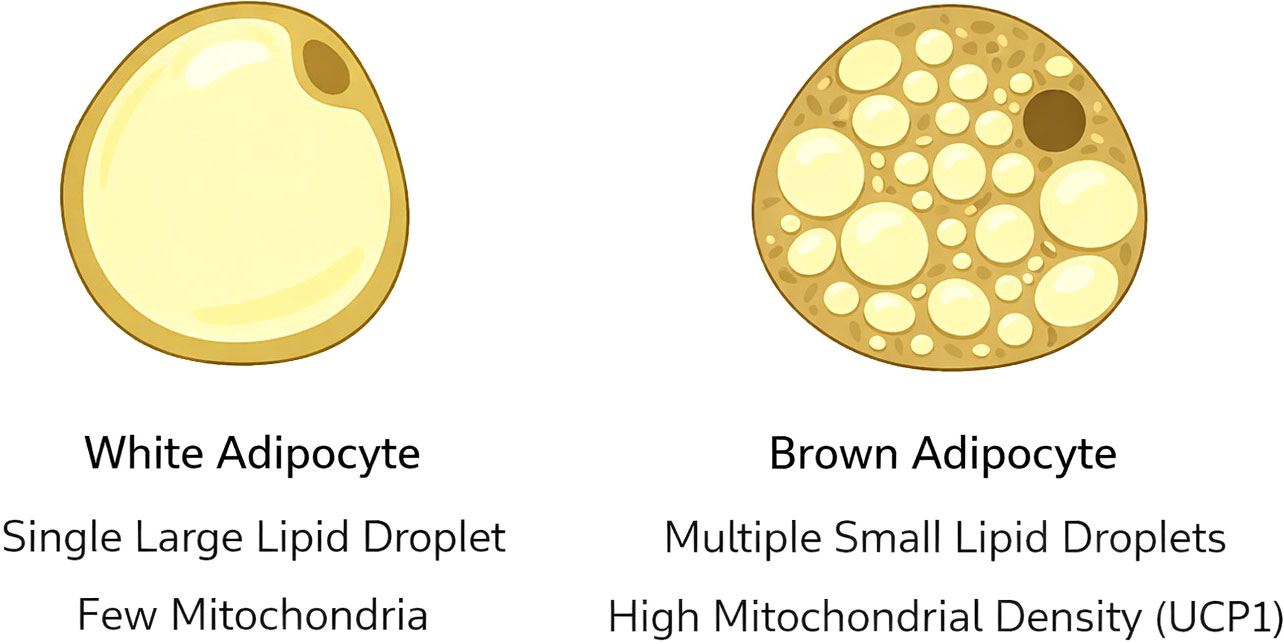
Structural comparison of white and brown adipocytes. White adipocytes are characterized by a single large lipid droplet and relatively few mitochondria, whereas brown adipocytes contain multiple small lipid droplets and abundant mitochondria expressing uncoupling protein 1 (UCP1), enabling non-shivering thermogenesis.

WAT is the most abundant adipose tissue in humans. WAT cells contain large single, spherical lipid droplets, and fewer blood vessels than BAT cells, thus giving it its white appearance (**Figure 1**). The lipid droplet accounts for approximately 90% of cell volume and consists primarily of triglycerides (8). When the lipid droplet expands, the cytoplasm and nucleus are displaced toward the cell periphery, giving the adipocyte its characteristic unilocular appearance.

Compared with brown adipocytes, white adipocytes contain relatively few mitochondria and exhibit a lower degree of vascularization. Their primary function is energy storage and mobilization. When caloric intake exceeds metabolic demand, excess energy is stored in WAT in the form of triglycerides. During periods of energy deficiency, these triglycerides are hydrolyzed into free fatty acids and glycerol, which can be used as fuel by other tissues (9).

WAT is abundant throughout the body and exists in both subcutaneous and visceral depots. Subcutaneous adipose tissue lies under the skin and commonly occurs in the abdominal wall, hips, thighs, arms, and breast tissues. By contrast, visceral adipose tissue surrounds internal organs such as the liver, pancreas, kidneys, and heart (5).

In addition, WAT also has an endocrine function aside from energy storage (10). It aids in the release of hormones such as leptin and adiponectin, as well as inflammatory mediators that influence systemic metabolism. Excess visceral WAT has been strongly associated with an increased risk of metabolic disorders such as insulin resistance, type 2 diabetes, and cardiovascular disease.

### Brown adipose tissue

BAT differs both structurally and functionally from WAT. Brown adipocytes contain multiple small lipid droplets and a dense population of mitochondria. These mitochondria are rich in iron-containing cytochromes, which give the tissue its characteristic brown coloration (9).

BAT is highly vascularized and supplied by an extensive network of capillaries, reflecting its high metabolic activity and oxygen demand. It is also densely innervated by the sympathetic nervous system, which plays a central role in activating thermogenesis.

The distinctive characteristic of brown adipocytes is the presence of uncoupling protein 1 (UCP1), found in the inner mitochondrial membrane. Under normal conditions, mitochondria generate adenosine triphosphate (ATP) by using the proton gradient created during oxidative phosphorylation. In brown adipocytes, however, UCP1 uncouples this process. Instead of driving ATP synthesis, protons re-enter the mitochondrial matrix through UCP1, and the stored energy is released as heat (5).

**Figure 2:**
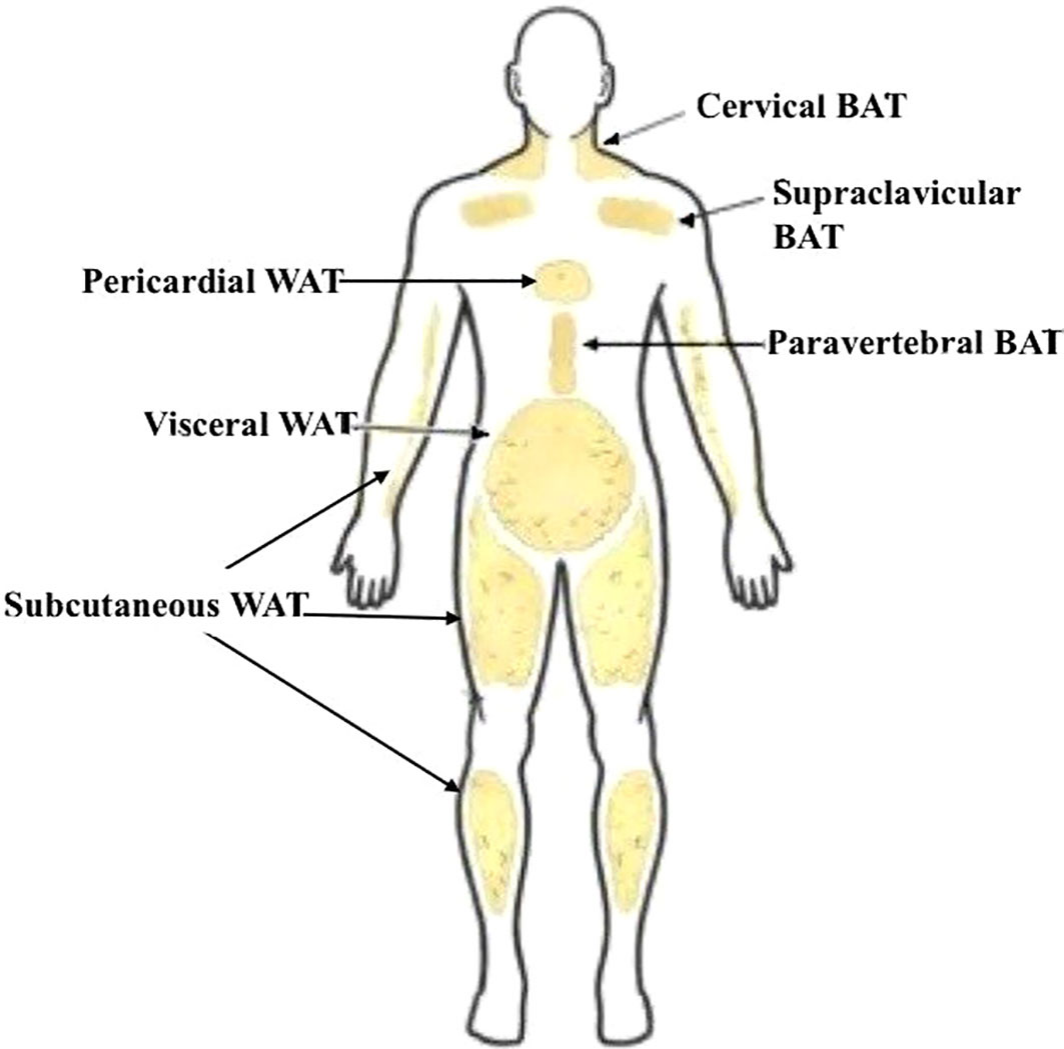
General anatomical distribution of white and brown adipose tissue. WAT is widely distributed in subcutaneous and visceral depots throughout the body. BAT in adult humans is primarily localized to the cervical, supraclavicular, and paravertebral regions, where it contributes to thermogenesis.

This thermogenic mechanism allows BAT to rapidly generate heat in response to cold exposure. In infants and small mammals, this function is critical for survival. Metabolically active BAT found in adults is typically located in the supraclavicular, cervical, paravertebral, and mediastinal regions (**Figure 2**). While the total volume of BAT in adults is less than in infants, it remains metabolically significant (2). In adults, BAT is localized to these key depots, where it contributes to thermoregulation. These areas are believed to function as localized heat sources that help maintain the temperature of blood supplied to vital organs (5).

### Developmental origins of WAT and BAT

Adipocytes originate from mesodermal precursor cells during embryonic development. Brown adipocytes appear earlier than white adipocytes and share a developmental lineage with skeletal muscle cells through Myf5-positive progenitor cells (11).

Transcriptional regulators such as PRDM16 play a central role in determining whether precursor cells differentiate into brown adipocytes or skeletal muscle cells. Loss of PRDM16 expression can lead to reduced brown fat differentiation and increased myogenic development, whereas increased PRDM16 expression promotes the formation of brown adipocytes (4).

Bone morphogenetic protein 7 (BMP7) has also been shown to stimulate brown adipocyte differentiation by inducing the expression of thermogenic genes such as UCP1 (12). These discoveries emphasize the importance of transcriptional regulation in adipose tissue evolution.

## Molecular regulation of brown adipocyte thermogenesis

Brown adipocyte thermogenesis is tightly regulated at the molecular level through coordinated transcriptional and signaling pathways. A central regulator of brown adipocyte identity is PR domain containing 16 (PRDM16), a transcriptional co-regulator that promotes brown fat differentiation while suppressing myogenic gene expression (13). PRDM16 interacts with PGC-1α (peroxisome proliferator-activated receptor gamma coactivator 1-alpha), a key driver of mitochondrial biogenesis and oxidative metabolism, thereby enhancing the thermogenic capacity of brown adipocytes (4).

**Figure 3:**
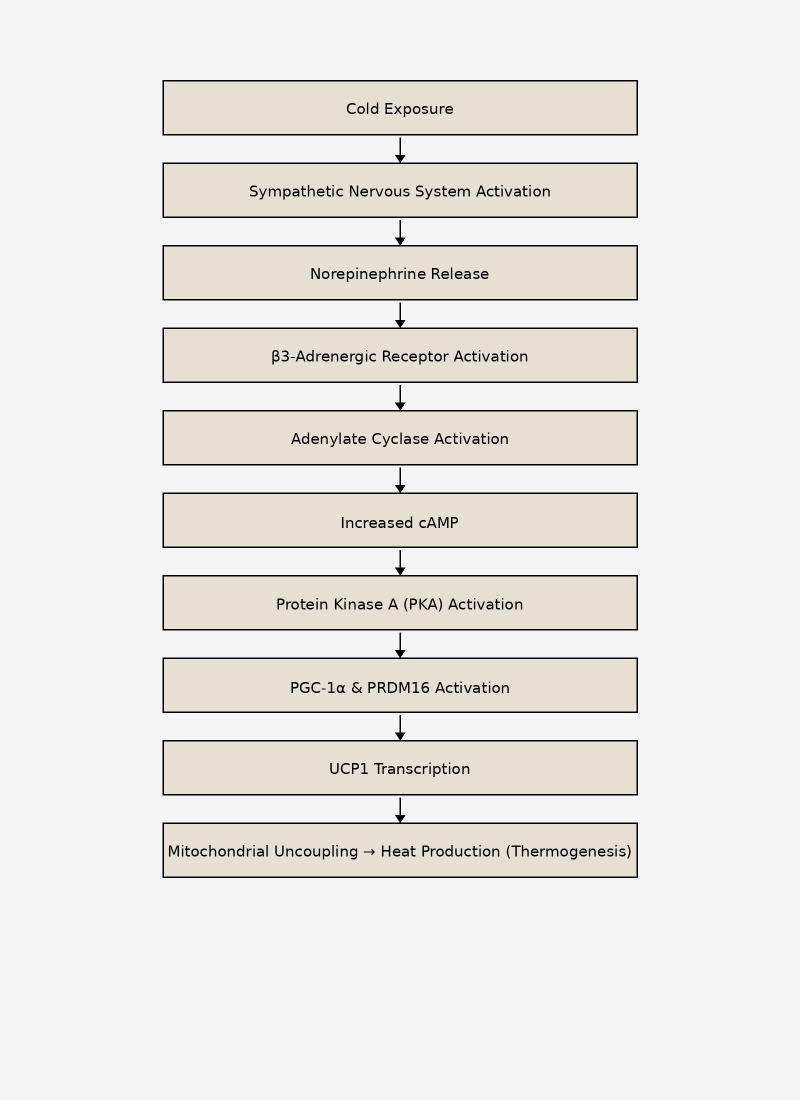
Molecular regulation of thermogenesis in brown adipocytes. Cold exposure activates the sympathetic nervous system, leading to norepinephrine release and β3-adrenergic receptor stimulation in brown adipocytes. Subsequent activation of the cAMP–protein kinase A signaling pathway promotes transcriptional regulation through PGC-1α and PRDM16, inducing UCP1 expression and mitochondrial uncoupling, resulting in heat production.

Sympathetic nervous system activation is the primary physiological trigger for thermogenesis. Cold exposure stimulates sympathetic nerve terminals to release norepinephrine, which binds to β3-adrenergic receptors on brown adipocytes. This activates adenylate cyclase, increases intracellular cyclic AMP (cAMP), and stimulates protein kinase A (PKA). The cAMP–PKA signaling cascade promotes lipolysis and enhances transcription of thermogenic genes, including UCP1. The resulting increase in mitochondrial uncoupling allows energy to be dissipated as heat rather than stored as ATP (**Figure 3**).

Bone morphogenetic protein 7 (BMP7) has been identified as an important factor in brown adipogenesis. BMP7 promotes commitment of precursor cells to a brown adipocyte lineage and enhances mitochondrial development (12). Collectively, these pathways demonstrate that BAT is not merely structurally distinct from WAT but is defined by a highly specialized regulatory network governing energy expenditure.

At the molecular level, white and BAT display distinct gene expression profiles that reflect their divergent roles. Brown adipocytes express genes associated with thermogenesis and mitochondrial biogenesis, including UCP1, PGC-1α, and PRDM16, which regulate both differentiation and functional activity (14). On the other hand, white adipocytes predominantly express genes involved in lipid storage and endocrine signaling, including leptin and the nuclear corepressor RIP140. These molecular distinctions underscore the opposing metabolic functions of the two adipose tissue types: WAT primarily stores energy, whereas BAT dissipates it in the form of heat (9).

## Beige adipocytes and adipose plasticity

Beige adipocytes represent a distinct, inducible thermogenic cell population that emerges within WAT depots under specific physiological conditions. Unlike classical brown adipocytes, which arise from a Myf5-positive lineage shared with skeletal muscle, beige adipocytes generally originate from Myf5-negative precursors. Their development is highly responsive to environmental stimuli, particularly chronic cold exposure and adrenergic activation (4).

The process of “browning” involves extensive transcriptional reprogramming and mitochondrial remodeling. Activation of β-adrenergic signaling stimulates expression of thermogenic genes, including UCP1, in otherwise white adipocytes (14). Importantly, beige adipocytes exhibit remarkable plasticity; their thermogenic phenotype can diminish once the stimulating signal is removed, suggesting reversible adaptation rather than permanent lineage conversion (15).

The understanding of beige adipocytes has evolved considerably in recent years. These cells arise within WAT depots in response to environmental and physiological stimuli such as cold exposure or β-adrenergic activation and contribute to adaptive thermogenesis under appropriate conditions (15). Recent molecular studies suggest that their recruitment is regulated through interactions among endocrine signaling, immune cell activity, and mitochondrial remodeling processes (16, 17), reinforcing the concept of adipose tissue as a highly dynamic and responsive organ. Collectively, these findings support the view that adipose tissue functions as a highly dynamic and responsive organ rather than a metabolically static storage site.

Adipose tissue plasticity has become a critical area of investigation since increasing the activation and recruitment of thermogenic adipocytes may increase energy expenditure and improve overall metabolic health (15).

Recent human studies suggest that beige adipocyte recruitment is more dynamic than previously believed. Wu et al. (15) reported that adult human supraclavicular depots contain a mixture of classical brown and beige adipocytes, suggesting functional heterogeneity. Furthermore, advances in single-cell transcriptomics have revealed that adipocyte lineage commitment is influenced by local microenvironmental factors and sympathetic tone, highlighting the complexity of adipose tissue remodeling (18).

Key structural and functional differences among white, brown, and beige adipocytes are summarized in **Table 1**.

**Table 1:** Comparative characteristics of white, brown, and beige adipocytes.

Feature	WAT	BAT	Beige
Developmental Origin	Mesenchymal stem cells	Myf5^+^ lineage	Myf5^−^ inducible precursors
Lipid Droplets	Single (unilocular)	Multiple (multilocular)	Multiple (inducible)
Mitochondrial Density	Low	High	Moderate to High
UCP1 Expression	Absent	Constitutive	Inducible
Primary Function	Energy storage	Thermogenesis	Adaptive thermogenesis
Sympathetic Innervation	Limited	Dense	Inducible
Plasticity	Low	Stable phenotype	Highly plastic

## Imaging and assessment techniques

### PET-CT Imaging

Since brown adipocytes are metabolically active and consume large amounts of glucose, they can be identified using fluorodeoxyglucose positron emission tomography combined with computed tomography (FDG PET-CT). This imaging method identifies regions showing increased glucose uptake relative to active BAT areas. Also, studies in adult humans have shown that exposure to cold significantly increases BAT activity and glucose uptake, confirming the presence of functioning BAT (5).

## MRI-based techniques

Magnetic resonance imaging (MRI) offers a noninvasive method for the detection of BAT without exposure to ionizing radiation. MRI techniques take advantage of differences in water-to-fat ratios between BAT and WAT, since BAT is more vascularized and contains more water. Differentiation between BAT and WAT based on their unique physiological characteristics, particularly through water-fat separation and Dixon imaging techniques, is possible (19).

Advances in recent imaging techniques have improved the quantification of BAT outside the scope of glucose uptake alone. Chen et al. (20) explained that using a combination of PET/MRI methods allows for a more distinct differentiation between active BAT and inactive adipose tissue areas by combining metabolic and structural imaging markers. These refinements improve the reliability of BAT assessment and may enhance future interventional studies evaluating thermogenic activation.

In addition to established imaging modalities such as PET-CT and MRI, emerging techniques including optical imaging have been explored for the assessment of BAT. Optical imaging approaches, particularly near-infrared fluorescence imaging, offer potential advantages such as high sensitivity and the ability to visualize metabolic activity at the tissue level. Although still primarily used in preclinical and experimental settings, these methods may provide valuable complementary insights into BAT function and activation in future clinical applications (21).

## Clinical and translational implications of thermogenic adipose tissue

The detection of metabolically active BAT in adult humans is reinvigorating interest in its clinical significance. Imaging studies have demonstrated that those with higher BAT activity often show improved glucose handling and enhanced insulin sensitivity, which indicates that BAT can be involved in metabolic regulation in a meaningful way (6). Activation of BAT enhances glucose uptake and utilization of fats, suggesting that thermogenic adipose tissue may contribute to improved metabolic regulation under certain physiological circumstances (22).

Reduced BAT volume and diminished thermogenic responsiveness are frequently observed in individuals with obesity (23). This relationship has driven continued investigation into strategies aimed at enhancing BAT activity or promoting browning within WAT. Approaches including β3-adrenergic receptor agonists, controlled cold exposure, and lifestyle-based interventions have been explored, although reported outcomes have varied across studies and populations (24).

Although experimental evidence is promising, translating BAT activation into clinical therapy has proven challenging (25). The thermogenic effectiveness of BAT is modest in adult human beings, overall, relative to that reported in small mammals, and its influence in association with pharmacologic stimulation leads to questions of cardiovascular safety as well as long term effectiveness. Despite this, thermogenic adipose tissue remains a very interesting adjunct target in the prevention and treatment of metabolic disease, especially as an adjunct to standard lifestyle and nutritional interventions and remains a focus of considerable interest.

## Recent advances (2018–2024)

In recent years, there has been substantial progress in understanding the regulation and functional significance of thermogenic adipose tissue in humans. New advances in tools, imaging techniques, molecular biology, and clinical research have provided updated insights into the variability, activation, and therapeutic potential of brown and beige adipocytes (25).

Importantly, contemporary research has expanded beyond the traditional characterization of BAT as solely a thermogenic tissue. Emerging data indicate that BAT may also exert endocrine effects through the secretion of bioactive factors, often termed batokines, which influence systemic glucose metabolism, vascular function, and inflammatory signaling pathways (26, 27). This evolving perspective has intensified interest in therapeutic strategies aimed at safely enhancing BAT activity through pharmacologic, environmental, or behavioral interventions.

One of the most notable developments has been the recognition of significant inter-individual variability in BAT activity. Factors such as age, sex, body composition, and metabolic health appear to influence both the presence and responsiveness of BAT (28). Younger individuals and those with lower adiposity generally exhibit higher BAT activity, whereas aging and obesity are associated with reduced thermogenic capacity. In addition, sex-based differences have been reported, with some studies suggesting that females may demonstrate greater BAT activation under certain conditions, potentially reflecting hormonal influences on adipose tissue regulation (29).

Cold exposure remains one of the most effective physiological stimuli for BAT activation, and recent human studies have refined protocols for inducing thermogenesis through controlled environmental exposure. These investigations have demonstrated that repeated cold exposure can enhance BAT activity and promote the browning of WAT, although the magnitude and sustainability of these effects vary considerably among individuals (30). This variability underscores the importance of personalized approaches when considering therapeutic applications.

Pharmacologic strategies targeting thermogenic adipose tissue have also advanced in recent years and have received increasing attention. β3-adrenergic receptor agonists have shown potential in activating BAT and increasing energy expenditure; however, their effectiveness in humans has been inconsistent, partly due to differences in receptor distribution compared to rodent models. β3-adrenergic receptor agonists, such as mirabegron, have demonstrated the ability to activate BAT and increase energy expenditure in humans by stimulating cyclic AMP–dependent signaling pathways that enhance UCP1 expression and mitochondrial activity.

In addition to adrenergic stimulation, other emerging strategies include agents that modulate mitochondrial function and biogenesis, thyroid hormone signaling, endocrine signaling pathways, fibroblast growth factor pathways, and adipocyte differentiation, all of which influence thermogenic capacity. While some of these approaches have demonstrated promising metabolic effects (31), concerns regarding safety, specificity, cardiovascular risk, variability in responsiveness, and long-term efficacy remain important considerations. Continued investigation into targeted and tissue-specific activation of thermogenic adipocytes will be critical for translating these findings into viable therapeutic interventions (23, 32).

Advances in technology have aided in the improved ability to detect and characterize thermogenic adipose tissue. In addition to established imaging modalities such as PET-CT and MRI, emerging techniques—including optical imaging approaches—are being examined to provide enhanced sensitivity and spatial resolution in the assessment of BAT activity. These developments may provide a more precise evaluation of thermogenic function in both research and clinical settings.

Overall, these recent findings highlight the complexity and heterogeneity of thermogenic adipose tissue in humans. They also highlight that, while BAT activation represents a promising avenue for metabolic intervention, its clinical application will require a more refined understanding of individual variability, regulatory mechanisms, and long-term outcomes.

## Limitations and future directions

Despite substantial advances in the understanding of thermogenic adipose tissue, several important limitations and knowledge gaps remain. One of the primary challenges lies in the variability of BAT detection and quantification in humans. Imaging techniques such as PET-CT, while widely used, are influenced by environmental conditions, including temperature and recent dietary intake, which can affect the apparent activity of BAT (28). This variability complicates direct comparisons across studies and may contribute to inconsistent findings regarding the prevalence and functional significance of BAT in different populations.

Another key limitation is the incomplete understanding of the long-term effects of BAT activation. While acute stimulation of thermogenic adipose tissue has been shown to increase energy expenditure and improve certain metabolic parameters, it remains unclear whether sustained activation can produce clinically meaningful and durable outcomes in humans. Furthermore, the relative contribution of BAT to whole-body energy balance appears modest compared to that observed in small animal models, raising questions about its overall therapeutic impact.

Emerging research also highlights the need to better understand inter-individual variability in thermogenic adipose tissue activity. Factors such as age, sex, genetic background, and metabolic status appear to influence both BAT presence and responsiveness. However, the mechanisms underlying this variability are not yet fully defined. Improved characterization of these factors may enable more personalized approaches to therapeutic intervention.

Pharmacologic interventions to stimulate BAT and induce browning of WAT have shown promise but are associated with several challenges. β3-adrenergic receptor agonists, for example, have demonstrated variable efficacy in human studies and may carry potential cardiovascular risks with prolonged use. Additionally, differences in receptor distribution and sensitivity between humans and animal models complicate the translation of preclinical findings into clinical practice. Despite recent advances, translation into clinical therapy remains challenging. Pharmacologic stimulation of BAT using β3-adrenergic agonists has produced variable outcomes in human studies, likely reflecting species-specific differences in receptor distribution compared with rodent models (33). Questions surrounding long-term safety, sustainability of activation, and the overall magnitude of metabolic benefit remain unresolved. Nevertheless, the growing body of evidence supports an important contributory role for BAT and beige adipocytes in human metabolic regulation.

Future research should focus on combining advances in molecular biology, imaging techniques, and clinical investigations to determine a better role of thermogenic adipose tissue in human health. Longitudinal human studies are needed to assess the sustainability and safety of BAT activation strategies, while continued development of non-invasive imaging techniques may improve the accuracy of BAT assessment. Additionally, further exploration of endocrine signaling pathways and adipose tissue crosstalk may uncover novel targets for therapeutic intervention.

Addressing these limitations will be important for understanding and translating current knowledge of brown and beige adipose tissues into effective strategies for the treatment and prevention of obesity and other metabolic disorders.

## Conclusion

BAT and WAT fulfill fundamentally distinct but complementary functions in human physiology. While the main roles of WAT are energy storage and endocrine regulation, BAT increases energy expenditure using mitochondria and non-shivering thermogenesis. Understanding that metabolically active BAT persists in adult humans, along with the identification of inducible beige adipocytes in WAT depots, has substantially reshaped current understanding of adipose tissue as a dynamic and metabolically responsive organ rather than a passive energy storage tissue.

Regulatory principles that underline thermogenic adipocyte differentiation and activation—including sympathetic signaling pathways, transcriptional regulators (PRDM16 and PGC-1α), and mitochondrial remodeling processes—have been further elucidated by several studies in the field of molecular and cellular science. These results highlight the impressive plasticity of adipose tissue and its ability to adapt to environmental and physiological needs. More recently, findings supporting endocrine signaling via adipose-derived signaling molecules point toward the role of thermogenic fat depots acting at the systemic level rather than solely in heat production.

From a clinical perspective, increasing attention has focused on the potential of BAT activation and WAT browning as complementary strategies for improving metabolic health. Although translational challenges remain—including variability in human BAT activity and limitations of pharmacologic stimulation—the growing body of experimental and clinical data supports continued investigation into therapeutic approaches targeting thermogenic adipose tissue.

Continued progress in molecular biology, imaging technologies, and human metabolic studies has further deepened our understanding of adipose tissue biology. Research investigating the safe modulation of thermogenic adipocyte activity may lead to the development of alternative therapeutics for obesity, metabolic syndrome, and related cardiometabolic diseases. As knowledge of adipose tissue heterogeneity continues to expand, its role in metabolic regulation will likely remain a primary focus of both basic and translational research.

When taken together, current findings suggest that enhancing BAT activity or promoting browning within WAT may represent a complementary approach to addressing obesity and metabolic disease (10). Continued investigation integrating molecular biology, advanced imaging techniques, and carefully controlled human studies will be critical for translating these insights into clinically meaningful interventions.

## References

[1] Avram AS Avram MM James WD . Subcutaneous fat in normal and diseased states: 2. Anatomy and physiology of white and brown adipose tissue. J Am Acad Dermatol. (2005) 53:671–83. doi: 10.1016/j.jaad.2005.05.015, PMID: 16198791

[2] Forner F Kumar C Luber CA Fromme T Klingenspor M Mann M . Proteome differences between brown and white fat mitochondria reveal specialized metabolic functions. Cell Metab. (2009) 10:324–35. doi: 10.1016/j.cmet.2009.08.014. PMID: 19808025

[3] Saely CH Geiger K Drexel H . Brown versus white adipose tissue: a mini-review. Gerontology. (2012) 58:15–23. doi: 10.1159/000321319, PMID: 21135534

[4] Seale P Bjork B Yang W Kajimura S Chin S Kuang S . PRDM16 controls a brown fat/skeletal muscle switch. Nature. (2008) 454:961–7. doi: 10.1038/nature07182. PMID: 18719582 PMC2583329

[5] Sacks H Symonds ME . Anatomical locations of human brown adipose tissue: functional relevance and implications in obesity and type 2 diabetes. Diabetes. (2013) 62:1783–90. doi: 10.2337/db12-1430. PMID: 23704519 PMC3661606

[6] Cypess AM Lehman S Williams G Tal I Rodman D Goldfine AB . Identification and importance of brown adipose tissue in adult humans. N Engl J Med. (2009) 360:1509–17. doi: 10.1056/NEJMoa0810780. PMID: 19357406 PMC2859951

[7] Van Marken Lichtenbelt WD Vanhommerig JW Smulders NM Drossaerts JM Kemerink GJ Bouvy ND . Cold-activated brown adipose tissue in healthy men. N Engl J Med. (2009) 360:1500–8. doi: 10.1056/nejmoa0808718. PMID: 19357405

[8] Shen W Wang Z Punyanita M Lei J Sinav A Kral JG . Adipose tissue quantification by imaging methods: a proposed classification. Obes Res. (2003) 11:5–16. doi: 10.1038/oby.2003.3. PMID: 12529479 PMC1894646

[9] Bargut TC Aguila MB Mandarim-de-Lacerda CA . Brown adipose tissue: updates in cellular and molecular biology. Tissue Cell. (2016) 48:452–60. doi: 10.1016/j.tice.2016.08.001. PMID: 27561621

[10] Tabei S Chamorro R Meyhöfer SM Wilms B . Metabolic effects of brown adipose tissue activity due to cold exposure in humans: a systematic review and meta-analysis of RCTs and Non-RCTs. Biomedicines. (2024) 12:537. doi: 10.3390/biomedicines12030537. PMID: 38540150 PMC10968636

[11] Perwitz N Wenzel J Wagner I Buning J Drenckhan M Zarse K . Cannabinoid type 1 receptor blockade induces transdifferentiation towards a brown fat phenotype in white adipocytes. Diabetes Obes Metab. (2010) 12:158–66. doi: 10.1111/j.1463-1326.2009.01133.x. PMID: 19895638

[12] Tseng YH Kokkotou E Schulz TJ Huang TL Winnay JN Taniguchi CM . New role of bone morphogenetic protein 7 in brown adipogenesis and energy expenditure. Nature. (2008) 454:1000–4. doi: 10.1038/nature07221. PMID: 18719589 PMC2745972

[13] Mao L Lu J Hou Y Nie T . Directly targeting PRDM16 in thermogenic adipose tissue to treat obesity and its related metabolic diseases. Front Endocrinol. (2024) 15:1458848. doi: 10.3389/fendo.2024.1458848. PMID: 39351529 PMC11439700

[14] Enerback S . The origins of brown adipose tissue. N Engl J Med. (2009) 360:2021–3. doi: 10.1056/NEJMcibr0809610. PMID: 19420373

[15] Wu J Boström P Sparks LM Ye L Choi JH Giang AH . Beige adipocytes are a distinct type of thermogenic fat cell in mouse and human. Cell. (2012) 150:366–76. doi: 10.1016/j.cell.2012.05.016. PMID: 22796012 PMC3402601

[16] Ikeda K Maretich P Kajimura S . The common and distinct features of brown and beige adipocytes. Trends Endocrinol Metab. (2018) 29:191–200. doi: 10.1016/j.tem.2018.01.001. PMID: 29366777 PMC5826798

[17] Wang W Seale P . Control of brown and beige fat development. Nat Rev Mol Cell Biol. (2016) 17:691–702. doi: 10.1038/nrm.2016.96. PMID: 27552974 PMC5627770

[18] Rajbhandari P Arneson D Hart SK Ahn IS Diamante G Santos LC . Single cell analysis reveals immune cell–adipocyte crosstalk regulating the transcription of thermogenic adipocytes. Cell Rep. (2019) 26:1–12. doi: 10.7554/eLife.49501, PMID: 31644425 PMC6837845

[19] Nedergaard J Bengtsson T Cannon B . Unexpected evidence for active brown adipose tissue in adult humans. Am J Physiol Endocrinol Metab. (2007) 293:E444–52. doi: 10.1152/ajpendo.00691.2006. PMID: 17473055

[20] Chen KY Cypess AM Laughlin MR Haft CR Hu HH Bredella MA . Brown adipose reporting criteria in imaging studies (BARCIST 1.0): recommendations for standardized FDG-PET/CT experiments in humans. Cell Metab. (2016) 24:210–22. doi: 10.1016/j.cmet.2016.07.014, PMID: 27508870 PMC4981083

[21] Liu J Ni L Peng M Liang Y Lu C Zheng H . Optical imaging for brown or beige adipose tissue. VIEW. (2024) 5:20240022. doi: 10.1002/VIW.20240022. PMID: 41925065

[22] Blondin DP Tingelstad HC Noll C Frisch F Phoenix S Guérin B . Dietary fatty acid metabolism of brown adipose tissue in cold-acclimated men. Nat Commun. (2017) 8:14146. doi: 10.1038/ncomms14146. PMID: 28134339 PMC5290270

[23] Carpentier AC Blondin DP Haman F Richard D . Brown adipose tissue: a translational perspective. Endocr Rev. (2023) 44:143–92. doi: 10.1210/endrev/bnac015. PMID: 35640259 PMC9985413

[24] Leitner BP Huang S Brychta RJ Duckworth CJ Baskin AS McGehee S . Mapping of human brown adipose tissue in lean and obese young men. Proc Natl Acad Sci USA. (2020) 114:8649–54. doi: 10.1073/pnas.1705287114. PMID: 28739898 PMC5559032

[25] Hachemi I U-Din M . Brown adipose tissue: activation and metabolism in humans. Endocrinol Metab (Seoul). (2023) 38:214–22. doi: 10.3803/EnM.2023.1659. PMID: 36972706 PMC10164504

[26] Villarroya F Cereijo R Villarroya J Giralt M . Brown adipose tissue as a secretory organ. Nat Rev Endocrinol. (2017) 13:26–35. doi: 10.1038/nrendo.2016.136. PMID: 27616452

[27] Scheja L Heeren J . The endocrine function of brown adipose tissues in health and cardiometabolic disease. Nat Rev Endocrinol. (2019) 15:507–24. doi: 10.1038/s41574-019-0230-6, PMID: 31296970

[28] Carpentier AC Blondin DP . Is stimulation of browning of human adipose tissue a relevant therapeutic target? Ann Endocrinol (Paris). (2024) 85:184–9. doi: 10.1016/j.ando.2024.05.006. PMID: 38871497

[29] Gavalda-Navarro A Villarroya J Cereijo R Giralt M Villarroya F . The endocrine role of brown adipose tissue: an update on actors and actions. Rev Endocr Metab Disord. (2022) 23:31–41. doi: 10.1007/s11154-021-09640-6. PMID: 33712997

[30] Rosell M Kaforou M Frontini A Okolo A Chan YW Nikolopoulou E . Brown and white adipose tissues: intrinsic differences in gene expression and response to cold exposure in mice. Am J Physiol Endocrinol Metab. (2014) 306:E945–64. doi: 10.1152/ajpendo.00473.2013. PMID: 24549398 PMC3989735

[31] Warner A Mittag J . Thyroid hormone and the central control of metabolism. J Mol Endocrinol. (2016) 56:R17–32. doi: 10.1530/jme-12-0068. PMID: 22586142

[32] Cypess AM Weiner LS Roberts-Toler C Franquet EE Kessler SH Kahn PA . Activation of human brown adipose tissue by a β3-adrenergic receptor agonist. Cell Metab. (2015) 21:33–8. doi: 10.1016/j.cmet.2014.12.009. PMID: 25565203 PMC4298351

[33] Cypess AM Cannon B Nedergaard J Kazak L Chang DC Krakoff J . Emerging debates and resolutions in brown adipose tissue research. Cell Metab. (2025) 37:12–33. doi: 10.1016/j.cmet.2024.11.002. PMID: 39644896 PMC11710994

